# Decadal Trends in Ambient Air Pollutants and Their Association with COPD and Lung Cancer in Upper Northern Thailand: 2013–2022

**DOI:** 10.3390/toxics12050321

**Published:** 2024-04-28

**Authors:** Pachara Sapbamrer, Pheerasak Assavanopakun, Jinjuta Panumasvivat

**Affiliations:** 1 Department of Academic, Montfort College, Chiang Mai 50000, Thailand; 2 Department of Community Medicine, Faculty of Medicine, Chiang Mai University, Chiang Mai 50200, Thailandpheerasak.assava@cmu.ac.th (P.A.); 3 Environmental and Occupational Medicine Excellence Center (EnOMEC), Faculty of Medicine, Chiang Mai University, Chiang Mai 50200, Thailand

**Keywords:** air pollution, particulate matter, respiratory disease, lung cancer, chronic obstructive pulmonary disease

## Abstract

Air pollution in upper northern Thailand raises health concerns. This study examined trends and associations between air pollutants and respiratory diseases, focusing on COPD and lung cancer during haze (December–May) and non-haze (June–November) seasons in upper northern Thailand from 2013 to 2022. This study utilized data from the Pollution Control Department and Chiang Mai Provincial Public Health. The key air pollutants included PM_10_, PM_2.5_, SO_2_, NO_2_, CO, and O_3_. Respiratory disease data included fatality rates for lung cancer and COPD and the re-admission rate for COPD. Results indicated peak air pollutant levels and COPD re-admission rates in March, with PM_2.5_ concentrations exceeding air quality standards from January to April. During haze periods, COPD fatality and re-admission rates significantly increased (mean difference: 0.43 and 4.23 per 1000-case population, respectively; *p* < 0.001), while lung cancer fatality rates were higher without statistical significance. Pearson correlation analysis found positive correlations between PM_10_, PM_2.5_, O_3_, and NO_2_ concentrations and COPD re-admission and fatality rates at 0–1 month lag times, with a declining trend observed at subsequent lag intervals of 2 to 3 months. Overall, this study highlights the predictable pattern of air pollution in the region, correlating with higher COPD fatality and re-admission rates.

## 1. Introduction

Air pollution is a major global environmental risk to population health. The World Health Organization (WHO) suggests that ninety-nine percent of the global population breathes air that exceeds the WHO guideline limits, and the population in low- and middle-income countries suffers from the highest exposures [1]. In Thailand, the upper northern region is the major area that faces a problem of air pollution, particularly particulate matter (PM) [2]. Biomass burning, climatic conditions, and topography are the main causes of air pollution in upper northern Thailand. The open burning of crop residues and forest fires during the dry season from January to April are primary sources of air quality in northern Thailand [3]. Forest fires occurring in neighboring countries can also transport air pollutants across Thailand [4]. Regarding the season pattern, the dry season, with low rainfall, low wind speed, and temperature inversion, occurs during November and March every year [5]. El Niño and La Niña are climate cycles that also have an impact on pollution levels. A study showed that the levels of PM_2.5_, carbon, and metal components during the haze season in El Niño years were significantly higher than in La Niña years due to differences in climatic conditions and other related meteorological factors [6]. Furthermore, upper northern Thailand has a mountain valley topography [7,8]. Therefore, when open burning and forest fires occur, certain air pollutants are trapped in the valleys, leading to escalating high concentrations of PM and haze smog during the dry season.

Exposure to ambient air pollutants such as PM, ozone (O_3_), sulfur dioxide (SO_2_), and nitrogen dioxide (NO_2_) contributes to various adverse health effects, particularly in the respiratory system [9]. Previous epidemiological studies suggest that exposure to PM and other gaseous pollutants increases the risk and fatality of respiratory diseases, such as chronic obstructive pulmonary disease (COPD) and lung cancer [9,10,11]. Air pollution contributes to the development of COPD by inducing inflammation of the airway and lung parenchyma through inflammatory cells (lymphocytes, neutrophils, and macrophages) and cytokines (tumor necrosis factor-α, interleukin-6, and interleukin-8), oxidative stress, and DNA damage [12,13]. In particular, PM_2.5_ is known as a human carcinogen in lung cancer due to its role in increasing inflammatory processes and causing alterations to microRNA and DNA methylation [14].

Therefore, the present study aimed to investigate the trends and associations between air pollutant levels and respiratory diseases, including the fatality rate of lung cancer and COPD, as well as the COPD re-admission rate during 2013–2022 in eight provinces of upper northern Thailand between the haze and non-haze seasons.

## 2. Materials and Methods

This study was conducted in eight provinces of upper northern Thailand, including Chiang Mai, Lam Phun, Lam Pang, Phrae, Nan, Phayao, Chiang Rai, and Mae Hong Son. The ten-year historical data on air pollution and respiratory diseases from 2013 to 2022 were collected using secondary data reports from the Air Quality Management Bureau, Pollution Control Department website [15], and the Health Regional Medical Office 1, Ministry of Public Health website [16].

**Figure 1:**
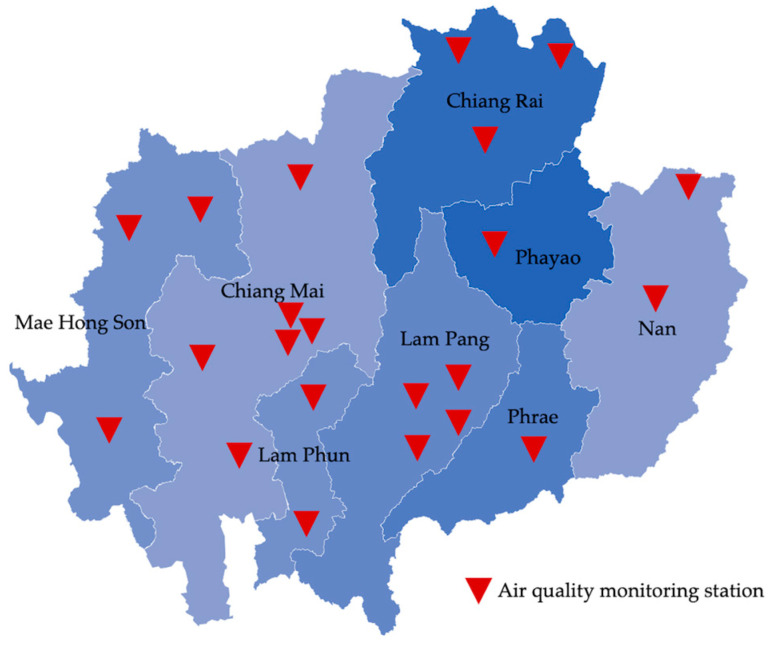
The number and location of air quality monitoring stations in upper northern Thailand.

The air pollutant data from the Pollution Control Department website [15], which the public provides and is free to access, were collected, including PM_10_–24 h, PM_2.5_–24 h, SO_2_–1 h, NO_2_–1 h, CO–1 h, and O_3_–1 h. Using standard limits on the air quality of each pollutant as the cutoff level. The total data were calculated as the mean and standard deviation. The total number of air quality monitoring stations was 22, distributed across 8 provinces. The details of the number and location of air quality monitoring stations in each province are identified in Figure 1.

The respiratory disease data were collected from the Health Regional Medical Office 1, Ministry of Public Health report on the website. The data indicated hospital in-patient cases from 99 public hospitals in upper northern Thailand [16], including re-admission cases for COPD, death cases from COPD and lung cancer, and the total number of in-patient cases for COPD and lung cancer. Lung cancer was identified by C34.0–34.3 and C34.8–34.9, and COPD was identified by J43–44. The data were extracted by months, years, and provinces, resulting in a total of 85,536 datasets. The total number of respiratory cases in the database was anonymized. The formula for calculating the fatality and re-admission rates was as follows:

Re-admission rate of COPD = (re-admission cases from COPD × 1000)/(COPD cases in inpatient department)

Fatality rate of COPD = (death cases from COPD × 1000)/(COPD cases in inpatient department)

Fatality rate of lung cancer = (death cases from lung cancer × 1000)/(lung cancer cases in inpatient department)

The dataset was categorized into two seasons, namely, the haze season (December–May) and the non-haze season (June–November). Data imputation using a regression model was used to retain the majority of the dataset’s data by substituting missing data with a different value (326 for SO_2_–1 h, 212 for NO_2_–1 h, 68 for CO–1 h, 53 for O_3_–1 h, 30 for PM_10_–24 h, and 387 for PM_2.5_–24 h). A comparative analysis of central tendency measures, such as mean and median, was conducted to ascertain the consistency of the imputed dataset with the original. The examination revealed marginal disparities in the values of each parameter between the imputed and unaltered datasets. Thus affirming the fidelity of the data imputation technique and its negligible influence on subsequent analytical outcomes. Additionally, associations between air pollutant levels and respiratory outcomes were examined across both the imputed and original datasets to comprehensively evaluate the implications of imputation on analytical outcomes. Therefore, the findings mirrored a consistent directionality across both datasets, reinforcing the reliability and applicability of the imputed dataset for investigative pursuits.

An independent *t*-test was utilized to compare the differences in air pollutant levels, fatality rates of lung cancer and COPD, and re-admission rates of COPD between the haze and non-haze seasons. Pearson correlation coefficient analysis was used to investigate the associations of air pollutant levels with the fatality rate of lung cancer and COPD and the re-admission rate of COPD across varying lag intervals, including lag 0 months, lag 1 month, lag 2 months, and lag 3 months. A dataset comprising 960 observations was conducted to analyze Pearson correlation coefficients based on monthly, yearly, and provincial values. The significance level was set at a *p*-value < 0.05.

This study was approved by the Research Ethics Committee of the Faculty of Medicine, Chiang Mai University (Study Code: COM-2566-0249).

## 3. Results

### 3.1. Air Pollutant Levels and Respiratory Diseases in Upper Northern Thailand during 2013–2022

During 2013–2022, an average of 27.53 ± 24.08 µg/m^3^ was detected for PM_2.5_–24 h and 42.02 ± 29.45 µg/m^3^ for PM_10_–24 h. Regarding gaseous pollutants, the average was 10.9 ± 0.92 ppb for SO_2_–1 h, 0.47 ± 4.69 ppb for NO_2_–1 h, 0.56 ± 0.39 ppm for CO–1 h, and 23.20 ± 10.87 ppb for O_3_–1 h. Considering respiratory diseases during 2013–2021, the data showed the average lung cancer fatality rate/1000 was 6.95 ± 5.49, whereas 80.54 ± 60.54 for COPD fatality rate/1000, and 16.16 ± 4.57 for COPD re-admission rate/1000. Additional air pollutant levels and the prevalence of respiratory diseases in each province are shown in Table 1. 

### 3.2. Trend of Air Pollutant Levels and Respiratory Diseases Classified by Month, 2013–2022

**Figure 2:**
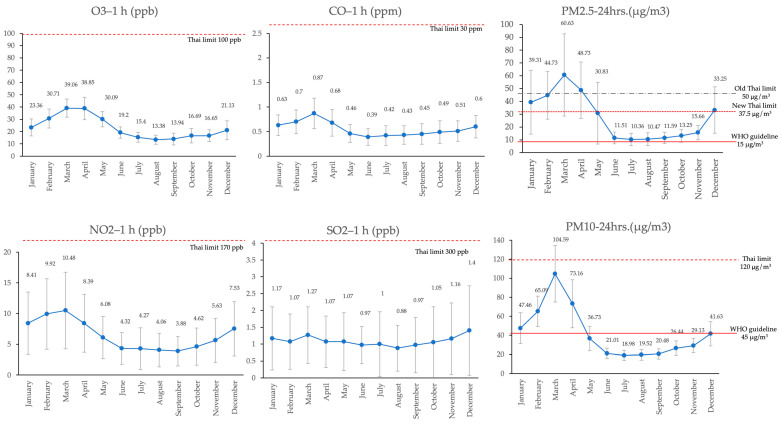
Trend of air pollution levels classified by month, 2013–2022. The red lines and dashed lines are indicated the standard level of each pollutants.

The monthly air pollutant levels from 2013 to 2022 are presented in Figure 2. The highest levels of PM_2.5_–24 h, PM_10_–24 h, NO_2_–1 h, CO–1 h, and O_3_–1 h were detected in March. Meanwhile, the highest levels of SO_2_ at 1 h were detected in December. The lowest levels of air pollutants varied from June to September, with most of the lowest levels found in July and August. When comparing PM_2.5_ and PM_10_ levels with WHO guidelines and Thai standard limits, PM_2.5_ levels during January–May and November and December exceeded the WHO guidelines, whereas PM_2.5_ levels during January and April exceeded the new standard limits of Thailand. PM_10_ levels during January–April exceeded the WHO guidelines. However, PM_10_ levels for all months did not exceed the Thai limit. SO_2_, NO_2_, CO, and O_3_ levels in all months did not exceed the Thai standard limits. The trend of air pollution, when analyzed separately in each province, also exhibited a similar pattern, with peaks for all pollutants occurring from March to April, except for SO_2_ (Figure S1).

**Figure 3:**
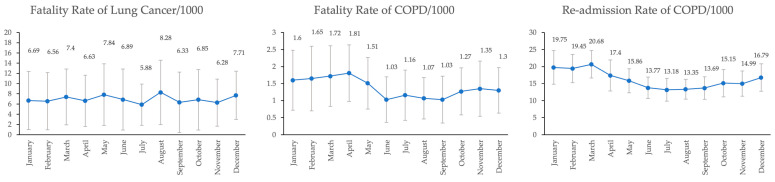
Trend of respiratory diseases classified by month, 2013–2022.

Respiratory diseases classified by month, 2013–2022, are presented in Figure 3. For COPD, the highest fatality rate was found in April, with an average of 1.81 per 1000 populations, whereas the highest re-admission rate was found in March, with an average of 20.68 per 1000 populations. The trend for the COPD fatality rate and re-admission rate had an upward trend during January and April. In contrast to lung cancer, the highest fatality rate was found in August, with an average of 8.28 per 1000 populations, and the trend of the fatality rate of lung cancer fluctuated.

### 3.3. Comparison of Air Pollutant Levels and Respiratory Diseases between Haze and Non-Haze Seasons

**Figure 4:**
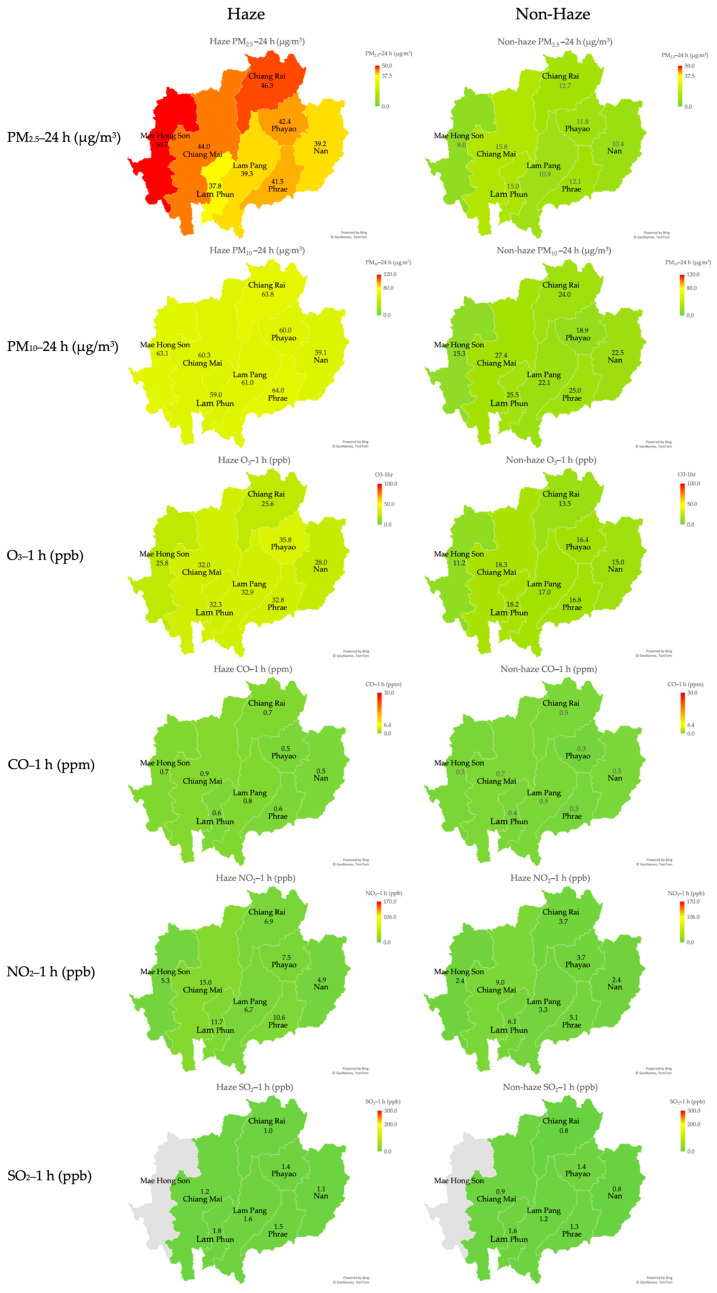
Visualization of air pollutants in eight provinces of upper northern Thailand between the haze and non-haze seasons during 2013–2022.

Visualization of air pollutants in eight provinces of upper northern Thailand between haze and non-haze seasons is presented in Figure 4. The levels of PM_2.5_, PM_10_, and O_3_ in all provinces during the haze season were higher than in the non-haze season. The highest PM_2.5_ levels during haze season were found in Mae Hong Son (50.7 µg/m^3^), Chiang Rai (46.3 µg/m^3^), and Chiang Mai (44.0 µg/m^3^), respectively. However, the levels of other pollutants, which included SO_2_, NO_2_, and CO, among eight provinces were rather similar both in haze and non-haze seasons. When comparing air pollutant levels and respiratory diseases between haze and non-haze seasons by using an independent sample *t*-test, the results found that the levels of all parameters of air pollutants during haze season were significantly higher than those during non-haze season (*p*-value < 0.01) (Table 2).

**Figure 5:**
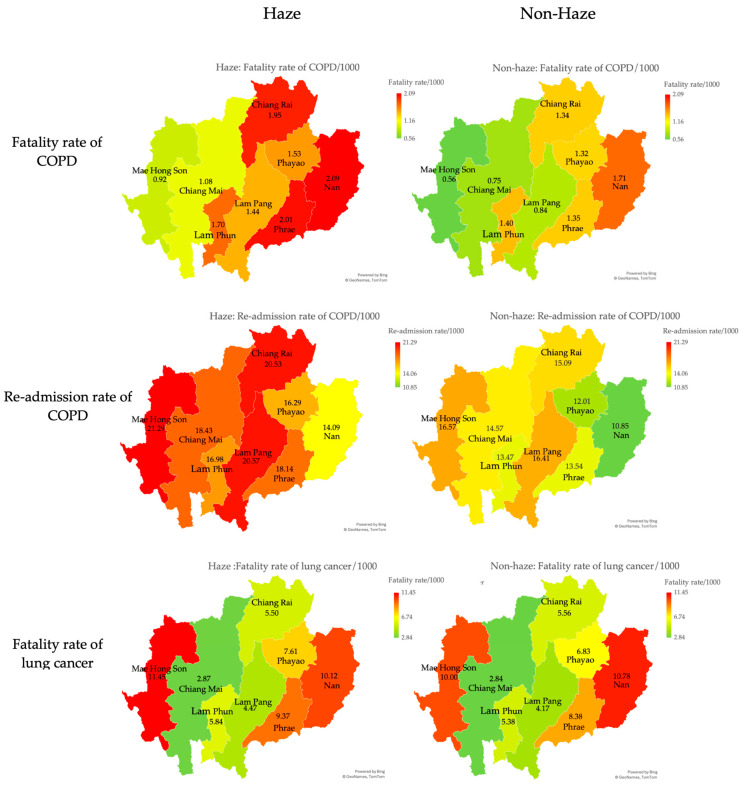
Visualization of respiratory diseases in eight provinces of upper northern Thailand between the haze and non-haze seasons during 2013–2022.

Fatality and re-admission rates of COPD in all provinces during the haze season were higher than in the non-haze season. Regarding respiratory diseases, fatality and re-admission rates of COPD during the haze season were significantly higher than those during the non-haze season (*p*-value < 0.01). However, there was no difference in the fatality rate of lung cancer during the haze and non-haze seasons (Table 2). Further details of respiratory diseases in eight provinces of upper northern Thailand between the haze and non-haze seasons are presented in Figure 5.

### 3.4. The Association of Air Pollutant Levels with Fatality Rates of COPD and Lung Cancer and Re-Admission Rates of COPD

The results found that the fatality rate of COPD had a weakly positive association with PM_2.5_, PM_10_, SO_2_, NO_2_, and O_3_ (*p*-value < 0.01). The re-admission rate of COPD showed a moderately positive association with PM_2.5_, PM_10_, NO_2_, CO, and O_3_ (*p*-value < 0.01). Regarding the fatality of lung cancer, there were no associations with PM_2.5_ and PM_10_, but there were weakly negative associations with SO_2_, NO_2_, and CO. At a lag interval of 1 month, a notably higher association was observed between the levels of PM_2.5_, CO, and O_3_ compared to the lag interval of 0 months. However, this association demonstrated a declining trend at subsequent lag intervals of 1 and 2 months. Particularly noteworthy was the declining trend observed in the association with PM_10_ levels. Additional associations are shown in Table 3.

## 4. Discussion

The air pollution in the northern part of Thailand followed a predictable seasonal pattern, occurring consistently throughout the same period each year [3,17]. The causes of air pollution in this region involve a multitude of aspects, including pollution sources, weather conditions, and atmospheric conditions. The chemical composition of PM_2.5_ might change depending on meteorological circumstances and emission sources, resulting in varied adverse health effects in different regions [18]. Previous studies showed that meteorological parameters consisting of solar radiation, humidity, and temperature can affect air pollution or health outcomes [19,20,21]. Temperature inversion exacerbated pollutant levels, particularly PM_2.5_ concentrations, and sunlight duration had an influence on O_3_ [20,21]. However, this study did not include these as confounding variables due to database limitations. When reviewing the provincial level, it was discovered that the problem is particularly noticeable in Mae Hong Son and Chiang Rai. Both provinces encounter border-related issues, suggesting a cross-border influence [22,23].

In June 2023, Thailand established an updated acceptable PM_2.5_ standard limit, which, however, remains higher than the level set by the World Health Organization. The existing WHO guidelines indicate that the average yearly levels of PM_2.5_ should not surpass 5 µg/m^3^. Additionally, the average daily exposure should not exceed 15 µg/m^3^ for more than three to four days per year [24]. However, Thailand’s recent announcement stated that the annual average concentrations of PM_2.5_ should not exceed 15 µg/m^3^, and the 24 h average exposures should not exceed 37.5 µg/m^3^ [25]. It suggests that the previous efforts to manage air pollution in this area were not efficient. Hence, it may be important to implement enhanced measures to deal with the root cause of pollution.

COPD is known for its acute onset, exacerbated by air pollutants. This study revealed a correlation between COPD and ambient air pollution levels. Exposure to pollutants was notably associated with increased hospital re-admissions and fatalities in COPD cases, and the trend of COPD cases aligned with the levels of air pollutants. Research conducted globally consistently indicates that air pollution significantly influences respiratory diseases in both short-term [26,27,28] and long-term exposures [29,30], leading to increased hospital admissions and mortality rates. Earlier research revealed a link between COPD hospitalizations and air pollution levels, especially concerning PM_2.5_, PM_10_, O_3_, and NO_2_ [26,27,29]. Exposure to PM_10_ and PM_2.5_ has been positively linked to an increased risk of respiratory mortality, ranging from 1.0073 to 1.023 [10,28]. Findings from a multicenter cohort project study on long-term exposure revealed that even exposure to concentrations less than 20 μg/m^3^ led to a rise in mortality rates [30]. In line with our research, studies conducted in northern Thailand have revealed associations between PM_2.5_, PM_10_, and O_3_ with COPD [31], leading to increased acute exacerbations and visits to the emergency room [32,33]. The increased rates of COPD re-admission and fatality may be attributed to the impact of PM on pulmonary function [34,35], specifically focusing on forced expiratory volume in one second (FEV_1_), a primary indicator of COPD fatality. As a result, heightened exposure to PM_2.5,_ which decreases FEV_1_ values, results in adverse outcomes, contributing to increased hospitalization and fatality. Exposure to pollution could trigger acute respiratory disease, with symptoms potentially worsening after a lag time. Immediate exposure to pollution was associated with COPD hospitalizations within a 0–7-day lag period [36,37,38]. Interestingly, our study found a positive correlation between pollutant concentrations and COPD fatality and re-admission rate at a 1-month lag time, suggesting that the effects of exposure could extend for up to a month. Consequently, it is advisable to conduct short-term health monitoring for at least one month after the haze season has passed.

During haze, there was an observed increase in the fatality rate for lung cancer; nevertheless, a statistically significant difference from the non-haze season was not found. Contrary to findings in other Thai studies, previous research indicated that the population-attributable fraction of PM_2.5_ for lung cancer was 16.8% in the Thai population [39]. Additionally, the reported number of deaths from lung cancer attributed to PM_2.5_ and PM_10_ in Northern Thailand was approximately 0.04% and 0.06%, respectively [40]. This could be clarified by considering that lung cancer has a prolonged period of exposure, leading to fatalities. Cumulative doses may accumulate over time before ultimately resulting in death. Based on a study conducted in the U.S. [41], the risk of lung cancer mortality increased from 1.13 to 1.33 times when transitioning from a 12-month moving average PM_2.5_ exposure to a 60-month moving average exposure. This study exclusively analyzed year-by-year mortality rates without considering cumulative effects, latency periods, or lag times.

PM contains toxic components such as heavy metals and polycyclic aromatic hydrocarbons (PAHs), which induce oxidative stress and DNA damage [12]. Specifically, high levels of benzo(a)pyrene (BaP), a type of PAH, are linked to deteriorated respiratory functions, increased morbidity and mortality of COPD, and an increased risk of lung cancer [42]. Research conducted in the upper northern region during haze season, primarily resulting from biomass burning and forest fires, shows elevated PAH levels. BaP was the main contributor to toxicity in the PM inhalation pathway [43].

This study demonstrated the predictable pattern of seasonal haze, which consistently exhibited an upward trend from January to April annually. These robust findings call for decisive action from stakeholders, including governmental bodies, environmental agencies, public health officials, and healthcare providers. Proactive measures are crucial for ensuring a well-prepared and timely public health response in anticipation of the upcoming haze season. The findings reveal the significant health impacts of air pollution on respiratory diseases. Consequently, it becomes imperative for authorities to thoroughly investigate the root causes of the pollution problems. Implementing policies or legal measures to target these root causes can pave the way for a more effective and lasting solution.

This study has a distinct strength in its focus on northern Thailand, an area globally recognized for its severe air pollution issues. Utilizing a decade-long dataset from a reliable standard source contributes to the robustness of our findings. The visualization of our data is not only comprehensive but also easily interpretable, offering valuable insights that can be seamlessly integrated into evidence-based decision-making processes and public health initiatives. However, this study has several limitations. Firstly, this study focused on the individual impact of each pollutant on the outcomes, potentially missing synergistic effects on the respiratory system where multiple pollutants may influence the same organ outcomes. Second, this study used a year-by-year cross-sectional approach with a 3-month lag time, which may not fully account for latency periods or longer lag times. Third, the number of air monitoring stations in each province was limited, which might not accurately reflect the exposure to pollutants across all areas. Lastly, constrained by the limitations of the health data reporting system, this study lacked comprehensive information, such as specific populations. Sub-analyses within specific groups, such as gender and age, were not conducted, potentially preventing the identification of distinct results. Additionally, database limitations prevented the inclusion of meteorological parameters as confounding variables in the study. In future studies, it is advisable to adopt a comprehensive approach by considering the combined impact of multiple pollutants on outcomes. Utilizing cohort designs, moving average calculations, or advanced time series analysis methods can enhance the understanding of associations. Furthermore, it may be necessary to access additional sources and data to acquire more records of variables, such as meteorological and population data, for a more comprehensive study, as this could involve integrating more influential factors into the analysis. Given the evident health impacts of air pollution and the predictability of seasonal haze, studies on reducing re-admissions and fatality rates are recommended.

## 5. Conclusions

The seasonal haze in northern Thailand follows a consistent trend from January to April, presenting a predictable pattern. This study established an association between air pollution, specifically PM_2.5_, PM_10_, and O_3_, and the fatality and re-admission rates of COPD. These findings emphasize the importance of an early and well-prepared public health response, especially before the haze season. Recommending the implementation of policies or legal measures to target the root causes of air pollution emerges as a crucial step toward a more effective and lasting solution.

## Figures and Tables

**Table 1:** Air pollutant levels and respiratory diseases in upper northern Thailand from 2013 to 2022.

Parameters	Upper Northern Region	Provinces							
		Chiang Mai	Lam Phun	Lam Pang	Phrae	Nan	Phayao	Chiang Rai	Mae Hong Son
Air pollution (duration 2013–2022), mean ± SD									
PM_2.5_–24 h (µg/m^3^)	27.53 ± 24.08	29.87 ± 20.92	26.31 ± 16.84	25.60 ± 20.02	28.83 ± 21.04	23.27 ± 20.42	24.92 ± 17.97	33.17 ± 33.91	28.11 ± 33.99
PM_10_–24 h (µg/m^3^)	42.02 ± 29.45	44.39 ± 25.12	42.25 ± 23.76	41.56 ± 27.23	44.52 ± 26.69	41.10 ± 27.78	39.36 ± 28.25	43.86 ± 33.62	39.26 ± 40.05
SO_2_–1 h (ppb)	10.9 ± 0.92	1.05 ± 0.42	1.73 ± 1.00	1.39 ± 0.52	1.33 ± 1.03	0.93 ± 0.87	1.40 ± 1.18	0.87 ± 0.34	N/A *
NO_2_–1 h (ppb)	0.47 ± 4.69	11.75 ± 5.60	8.93 ± 6.24	4.99 ± 2.28	8.00 ± 4.07	3.59 ± 1.94	5.32 ± 2.85	5.29 ± 3.22	3.69 ± 2.43
CO–1 h (ppm)	0.56 ± 0.39	0.75 ± 0.29	0.49 ± 0.22	0.64 ± 0.23	0.45 ± 0.20	0.45 ± 0.18	0.42 ± 0.19	0.63 ± 0.31	0.59 ± 0.26
O_3_–1 h (ppb)	23.20 ± 10.87	25.34 ± 9.73	25.47 ± 10.27	24.94 ± 10.90	24.77 ± 10.91	21.84 ± 9.62	25.11 ± 12.58	19.64 ± 8.92	18.92 ± 10.92
Respiratory outcome (duration 2013–2021), mean ± SD									
COPD									
death case	6.68 ± 4.94	10.83 ± 4.41	3.68 ± 2.35	7.48 ± 3.77	4.90 ± 2.43	8.24 ± 3.25	4.98 ± 2.84	12.50 ± 5.57	1.13 ± 1.05
fatality rate/1000	1.37 ± 0.81	0.91 ± 0.83	1.55 ± 0.94	1.14 ± 0.62	1.67 ± 0.84	1.90 ± 0.74	1.46 ± 0.74	1.64 ± 0.69	0.74 ± 0.68
re-admission case	80.54 ± 60.54	189.83 ± 41.68	36.54 ± 11.26	114.73 ± 37.54	45.68 ± 17.25	53.79 ± 19.01	45.48 ± 15.12	131.53 ± 37.99	27.51 ± 8.76
re-admission rate/1000	16.16 ± 4.57	16.48 ± 3.18	15.21 ± 1.18	18.47 ± 3.77	15.82 ± 4.64	12.37 ± 3.27	14.05 ± 3.61	17.78 ± 4.35	18.91 ± 5.27
Lung cancer									
death case	4.61 ± 3.25	7.86 ± 2.98	2.26 ± 1.46	5.47 ± 2.30	3.11 ± 2.06	6.62 ± 2.59	3.37 ± 1.91	7.28 ± 3.13	1.21 ± 1.10
fatality rate/1000	6.95 ± 5.49	2.85 ± 1.08	5.61 ± 3.68	4.31 ± 1.81	8.87 ± 5.35	10.58 ± 4.79	7.38 ± 3.56	5.53 ± 2.36	10.72 ± 9.94

* N/A = no data in 2013–2022.

**Table 2:** Comparison of air pollutant levels and respiratory diseases between the haze and non-haze seasons during 2013–2022.

Air Pollutants	Haze Season	Non-Haze Season	Mean Difference ± SE (95%CI)	*p*-Value ^+^
Air pollutants				
PM_2.5_–24 h (µg/m^3^)	42.91 ± 25.68	12.14 ± 5.20	30.77 ± 1.95 (28.42, 33.12)	<0.001 *
PM_10_–24 h (µg/m^3^)	61.44 ± 30.45	22.59 ± 7.27	38.85 ± 1.43 (36.05, 41.65)	<0.001 *
SO_2_–1 h (ppb)	1.18 ± 0.95	1.01 ± 0.88	0.17 ± 0.59 (0.05, 0.28)	0.004 *
NO_2_–1 h (ppb)	8.47 ± 5.20	4.46 ± 3.01	4.00 ± 0.27 (3.47, 4.54)	<0.001 *
CO–1 h (ppm)	0.66 ± 0.27	0.47 ± 0.45	0.21 ± 0.16 (0.18, 0.24)	<0.001 *
O_3_–1 h (ppb)	30.53 ± 10.18	15.88 ± 5.02	14.65 ± 0.52 (13.64, 15.67)	<0.001 *
Respiratory outcomes				
Fatality rate of COPD/1000	1.59 ± 0.84	1.16 ± 0.71	0.43 ± 0.06 (0.32, 0.54)	<0.001 *
Re-admission rate of COPD/1000	18.29 ± 4.55	14.06 ± 3.51	4.23 ± 0.29 (3.66, 4.79)	<0.001 *
Fatality rate of lung cancer/1000	7.15 ± 5.41	6.74 ± 5.57	0.41 ± 0.39 (−0.36, 1.18)	0.296

^+^ Independent *t*-test, * *p* < 0.05.

**Table 3:** Pearson correlation coefficient (*r*) of air pollutant levels with fatality rates of lung cancer and COPD and re-admission rates of COPD.

Parameters	Fatality Rate of COPD/1000				Re-Admission Rate of COPD/1000				Fatality Rate of Lung Cancer
	Lag 0 mo.	Lag 1 mo.	Lag 2 mo.	Lag 3 mo.	Lag 0 mo.	Lag 1 mo.	Lag 2 mo.	Lag 3 mo.	
PM_2.5_–24 h (µg/m^3^)	0.233 **	0.225 **	0.093 *	−0.061	0.446 **	0.499 **	0.428 **	0.170 **	−0.009
PM_10_–24 h (µg/m^3^)	0.288 **	0.225 **	0.095 **	−0.026	0.495 **	0.456 **	0.356 **	0.109 **	−0.004
SO_2_–1 h (ppb) ^1^	0.136 **	0.033	−0.051	−0.035	−0.048	0.058	0.044	0.008	−0.133 **
NO_2_–1 h (ppb)	0.171 **	0.077 *	−0.004	−0.091 *	0.315 **	0.328 **	0.227 **	0.073	−0.149 **
CO–1 h (ppm)	0.035	−0.026	−0.169 **	−0.216 **	0.308 **	0.444 **	0.317 **	0.150 **	−0.098 **
O_3_–1 h (ppb)	0.257 **	0.247 **	0.136 **	0.031	0.343 **	0.419 **	0.366 **	0.169 **	−0.051

^1^ The analysis was performed in seven provinces, excluding Mae Hong Son, due to missing data on SO_2_ levels. * = *p* < 0.05, ** = *p* < 0.001. mo. = months.

## Data Availability

The air pollution data are available as open data via the Pollution Control Department online data repository: http://air4thai.pcd.go.th (accessed on 23 August 2023). The anonymized data collected are available as open data via the Health Region 1, Ministry of Public Health online data repository (in Thai): https://cmi.ciorh1.com/web/index (accessed on 23 August 2023).

## Supplementary Materials

The following supporting information can be downloaded at: https://www.mdpi.com/article/10.3390/toxics12050321/s1: Figure S1: Trends of Air Pollutants in Eight Provinces of Upper Northern Thailand, during 2013–2022.
