# Supplementary figures and images for: Decadal Trends in Ambient Air Pollutants and Their Association with COPD and Lung Cancer in Upper Northern Thailand: 2013–2022
Source: Toxics. 2024 Apr 28;12(5):321. doi: 10.3390/toxics12050321 (PMC11125922; doi:10.3390/toxics12050321)

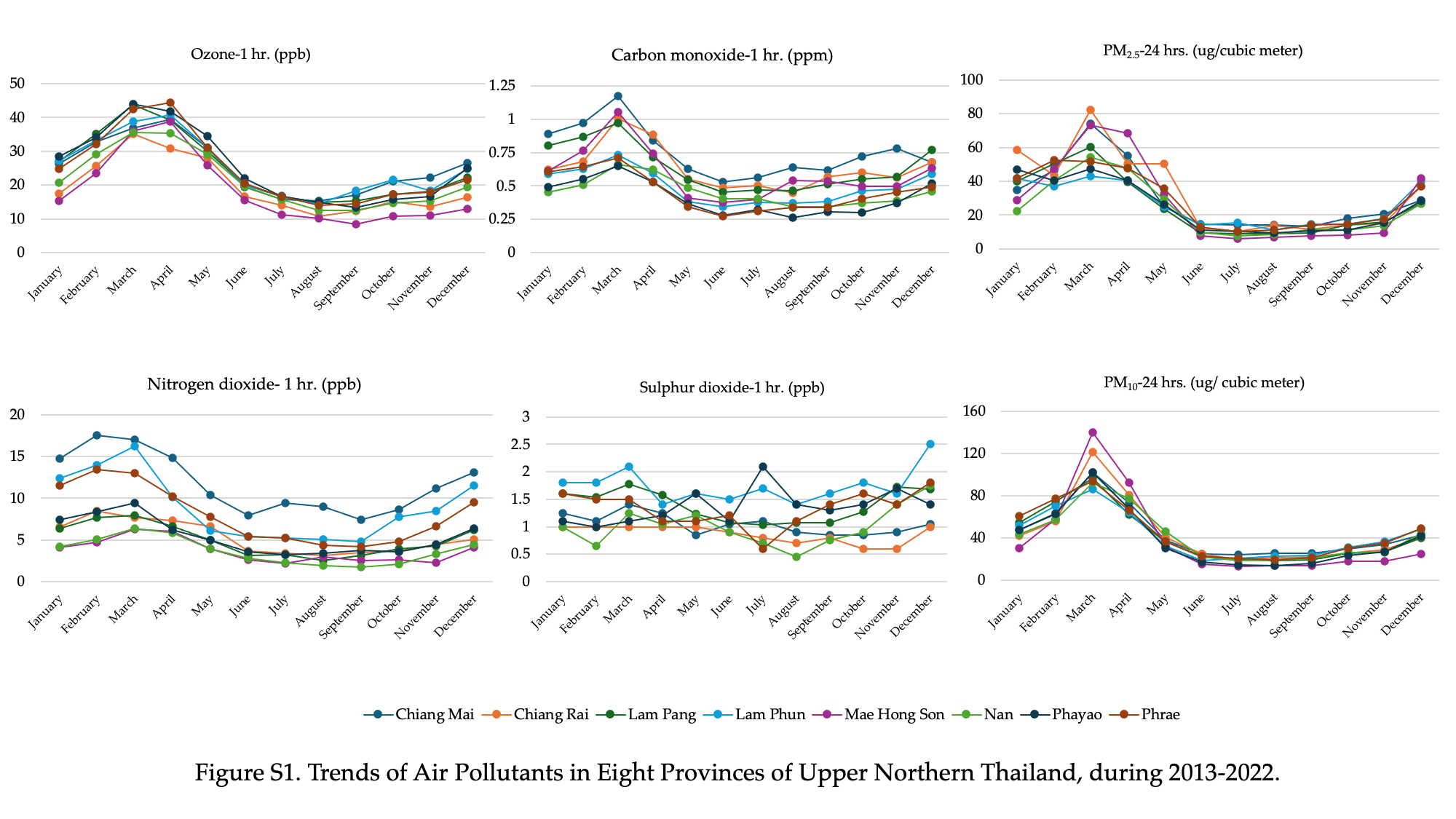

Supplement: Supplementary file 1 [file toxics-12-00321-s001.zip › toxics-2976152-supplementary.png]
